# 

**Published:** 1917-09

**Authors:** 


### CONTENTS FOR SEPTEMBER, 1917

### Original Articles

Some Practical Considerations in Chronic Heart Disease. By Arthur J. Patek, A. B., M. D., Milwaukee, Wis. .......... 45

A Resume of Things Concerning the Medical Officers' Training Camp at Fort Benjamin Harrison. By Samuel E. Earp, M. D., Indianapolis .......... 55

### Editorial

The Two Narcotic Laws .......... 58

Red Tape and Efficiency .......... 60

Surgical Bunglers .......... 61

Book Reviews .......... 62

Topics of Public Interest .......... 65

Society Meetings .......... 81

Our Contemporaries .......... 82

Obituary .......... 83

Personal .......... 84

Abstracts at end of original articles, on advertising pages and .......... 85

